# Mechanistic PBPK Modeling of Antibody–Drug Conjugates: Integration of Antibody Disposition, Linker Deconjugation, and Payload Pharmacokinetics

**DOI:** 10.3390/toxics14100931

**Published:** 2026-10-21

**Authors:** Kanchan Bharti, Yash Yogesh Gondegaonkar, Deepika Deepika, Vikas Kumar

**Affiliations:** Biomedical Research Institute of Southern Catalonia (IRBCatSud), Department of Chemical Engineering, Universitat Rovira I Virgili (URV), 43007 Tarragona, Spain; kanchan.bharti@irbcatsud.cat (K.B.); yashyogesh.gondegaonkar@estudiants.urv.cat (Y.Y.G.)

**Keywords:** antibody–drug conjugates, physiologically based pharmacokinetic modeling, payload pharmacokinetics, therapeutic proteins, drug-to-antibody ratio, linker deconjugation

## Abstract

Antibody–drug conjugates (ADCs) represent a rapidly advancing class of biological drugs used for cancer treatment. The integration of monoclonal antibodies, connected via chemical linkers, ensures the targeting potential of small-molecule payloads with cytotoxic potency. The United States Food and Drug Administration (USFDA) has announced its plan to phase out in vivo tests for monoclonal antibodies and other drugs with replacement from New Approach methodologies (NAMs) data. NAMs can include in vitro (cell-line and organoid testing), in silico modeling or other innovative approaches that have the capacity to predict the toxicity of compounds including ADCs. Among these proposed NAMs, physiologically based pharmacokinetic (PBPK) modeling has the capacity to provide a mechanistic framework capable of integrating antibody disposition, linker deconjugation dynamics, and payload small-molecule pharmacokinetics (PK). This review provides details about ADC and the developing ADC PBPK model, covering the tissue architecture, two-pore vascular transport, model hierarchy from minimal to whole-body model, parameterization strategies, regulatory scenario, applications, limitations and their future prospects.

## 1. Introduction

ADCs are an emerging class of biopharmaceuticals designed to treat cancer, comprising an active drug combined with targeted antibody via chemical linker, with the aim of attacking target cells and leaving behind healthy cells. Major advantages include targeted delivery of the drug payload, specificity, higher cytotoxic potency, and reduced multidrug resistance in cancer cells [1]. Multiple ADCs have been approved by the USFDA to date, mostly administered through the intravenous route. Despite their ability to specifically deliver the drug payload at the desired target by the virtue of conjugated antibody, ADCs suffer from the problem of having a very narrow therapeutic index. The ratio of the dose of a drug that causes toxicity in 50% of the population (TD_50_) to the dose that causes a therapeutic or effective response in 50% of the population (ED_50_) is known as the therapeutic index [2]. Hence, higher therapeutic values mean that the drug is safe and vice versa. Out of 55 ongoing studies on the clinical development of ADCs, 23 have been discontinued due to demonstration of a narrow therapeutic index [3]. A narrow therapeutic index constrains the dose that can be administered, with adverse effects frequently emerging before the ADC can achieve its maximum therapeutic efficacy. Additionally, these toxicities reduce the number of treatment cycles patients can tolerate and commonly lead to missed doses and dose reductions [3]. Another disadvantage of ADCs is poor in vivo distribution due to the presence of antibodies. The conjugated antibody tends to localize slowly at the targeted site, leading to an insufficient therapeutic level and heterogenous distribution in the tumor [4,5]. The overall quantity of antibodies accumulated within the tumor is a critical factor for optimization of the therapeutic index of ADCs. As mentioned above, many ADCs fail in clinical development because of their narrow therapeutic index, and regulatory agencies are encouraging NAMs in place of in vivo testing. Physiologically based pharmacokinetic (PBPK) modeling addresses the aforementioned limitations by providing a mechanistic organ-level framework that links each ADC analyte to the underlying physiology that governs its disposition [6]. The disposition of ADCs is described using many processes including neonatal Fc receptor (FcRn)-mediated recycling, target-mediated drug disposition (TMDD), passage of the antibody across the vascular and lymphatic compartment, and others. By integrating these processes, a PBPK model simulates the time course of intact ADC, total antibody, and free payload simultaneously across plasma, target tissues, and off-target tissues, including the tumor interstitium where the cytotoxic effect occurs. This mechanistic resolution enables PBPK to serve as a nonclinical method for the safety assessment of ADCs, supporting first-in-human dose projection, exposure–response analysis, and the design of dose adjustments in special populations. In this review, we describe the steps to develop a PBPK model for ADCs and discuss its applications in dose selection, DAR optimization, and linker–payload selection for improved efficacy and reduced toxicity, and utility for IVIVE. Particular emphasis is placed on exposure to characterization of ADC-related analytes and the safety assessment. The review is intended for pharmacometricians, translational and clinical pharmacologists, and toxicologists working on biologics drug development, as well as regulatory scientists and quantitative modelers seeking emerging opportunities and challenges for ADC PBPK modeling. Important parameters and their implementation, which differ from small-molecule drugs, are also highlighted in this review.

## 2. ADC Biology and Disposition

An ADC is constructed from three structurally distinct components: a tumor-targeting monoclonal antibody (mAb), a highly potent cytotoxic small-molecule payload, and a chemical linker that connects them (Figure 1). The mAb and the drug payload are assembled via conjugation chemistry that determines the resulting DAR and its distribution. Each of these components contributes a distinct set of physicochemical and biological properties to the final drug product, and their interplay defines both the therapeutic activity and the PK behavior of the ADC.

### 2.1. Structural Components: Antibody, Linker, and Payload

The antibody component of the ADCs comprises mABs, where the unique antigen target, once identified in the tumor, results in the binding of antigen–antibody on the surface of the cell. This mechanism ensures the targeting of the specific tumor cells through internalization of the ADCs and drug payload release via lysosomal processing [7,8]. The conjugation and internalization make the PK of ADC unique, with quantitative understanding required for different components, i.e., conjugate, total antibody and unconjugated payload. The antibody scaffold is a human Immunoglobulin G1 (IgG1) of approximately 150 kDa and occasionally an Immunoglobulin G4 (IgG4), where reduced effector function is preferred. Its variable region binds a tumor-associated cell-surface antigen with high affinity and specificity, while the conserved Fc region engages the FcRn in a pH-dependent manner, providing the salvage mechanism that gives IgG molecules their characteristic ~21-day plasma half-life. The size, charge, and Fc-mediated interactions of the antibody therefore demonstrate the slow lymph-flow-limited distribution into tissues and the long systemic circulation that distinguish ADCs from conventional small-molecule drugs [9,10,11]. Apart from human IgG-based ADCs, studies also have reported fused antihuman serum albumin nanobody to single-domain antibody–drug conjugates (sdADCs) targeting oncofetal antigen 5T4, which ultimately results in an optimum PK profile along with better tumor penetration and accumulation inside the tumor [12]. This indicates the importance of PK properties as the primary property that is affected by altering the protein scaffold in ADCs.

The payload is a highly potent cytotoxic small molecule, typically with sub-nanomolar to low-nanomolar half maximal inhibitory concentration (IC_50_) values, well below those of conventional chemotherapeutics. Common ADC payloads include tubulin inhibitors such as the auristatins MMAE and MMAF and the maytansinoids DM1 and DM4; DNA-damaging agents such as calicheamicin and pyrrolobenzodiazepine (PBD) dimers; and topoisomerase I inhibitors such as SN-38 (7-ethyl-10-hydroxycamptothecin) and the exatecan derivative DXd. Beyond potency, two payload properties are critical for in vivo behavior: membrane permeability, which determines whether the released payload remains in the target cell or diffuses to neighboring cells to drive a bystander effect; and lipophilicity, which when extreme can destabilize the conjugate, promote aggregation, and accelerate non-specific hepatic uptake [13,14].

The linker covalently joins the antibody to the payload and is the structural element most responsible for balancing systemic stability and intracellular release. Subsequent advances in linker chemistry, alongside a deeper understanding of therapeutic targets, paved the way for the development of second-generation ADCs. Linker stability affects off-target toxicity. When stability in circulation is inadequate, the payload can be released prematurely, exposing healthy tissues to cytotoxic agents and giving rise to systemic adverse effects. On the other hand, an overly stable linker can impede payload release within target cells, thereby diminishing the therapeutic effectiveness. Linkers fall into two broad categories: cleavable linkers, which are designed to release the payload in response to an intracellular trigger, and non-cleavable linkers, which release the payload only after complete proteolysis of the antibody backbone in the lysosome. Cleavable linkers include acid-labile chemistries (hydrazone, carbonate) cleaved at low lysosomal pH, reducible disulfides cleaved by intracellular glutathione, and peptide-based linkers (Val-Cit, Val-Ala, tetrapeptide glycylglycylphenylalanylglycine or GGFG) cleaved by lysosomal proteases such as cathepsin B. Non-cleavable linkers (most commonly the thioether Succinimidyl 4-(N-maleimidomethyl)cyclohexane-1-carboxylate) or SMCC) yield amino acid-payload adducts after antibody catabolism. Beyond cleavability, linker hydrophilicity is a primary determinant of ADME behavior: poorly hydrophilic linkers reduce conjugation efficiency, promote plasma aggregation, increase non-specific tissue uptake, and accelerate plasma clearance, particularly at high DAR [15,16,17]. A detailed table with information regarding the antibody, drug payload, liker, conjugation and DAR of the ADCs approved by USFDA is provided in Table 1.

### 2.2. DAR and Conjugation Chemistry

In ADCs, the DAR is a significant factor determining their cytotoxic potential. DAR refers to the average number of cytotoxic drug molecules attached to an individual antibody molecule. The value of DAR informs us about conjugated and unconjugated antibodies. DAR > 0 represents conjugated antibodies, i.e., where a cytotoxic payload is attached. The DAR value has a direct effect on therapeutic performance, as insufficient drug loading compromises potency, whereas excessively high drug loading can adversely affect toxicity profiles and PK behavior [19]. The fundamental challenge in linker design therefore lies in striking the right balance between stability and cleavability. Through careful optimization of bond types such as amide, disulfide, and carbon–nitrogen bonds alongside spatial configuration, it is possible to achieve both high plasma stability and controlled tumor-triggered cleavage. The molecular weight of the ADCs is very high, as it possesses resemblance to endogenous proteins. Apart from this, it also differs in structure, charge, hydrophobicity, binding affinity, potential for self-interaction, solubility, and colloidal suspension capacity [20,21]. Several factors govern their pharmacokinetic behavior: cellular uptake, target binding, payload potency, target penetration, and the absorption, distribution, metabolism and excretion (ADME) properties of the conjugate, which are largely dictated by the antibody. Crucially, an ADC is not a single-entity drug but a multi-component system in which the intact conjugate, total antibody, conjugated payload, and unconjugated (DAR = 0) payload all circulate simultaneously, and conjugation chemistry generates a heterogeneous mixture of species with different DARs that may exhibit distinct disposition. Conventional pharmacokinetic descriptions designed around a single small molecule or an unmodified antibody therefore cannot fully capture the disposition of ADCs, the dynamic interconversion among analytes, or the off-target exposure that drives systemic toxicity, particularly in patients with comorbidities requiring dose adjustment. However, this can be easily addressed by utilizing mathematical models such as PBPK.

Conjugation chemistry determines how many payload molecules are attached to each antibody and where on the antibody they are placed, with major implications for the resulting DAR distribution. Lysine-based conjugation is stochastic and yields a heterogeneous mixture of species ranging from DAR 0 to 8: T-DM1, for example, is conjugated through surface lysines and exhibits an average DAR close to 3.5 [22]. Cysteine-based conjugation, after partial reduction of inter-chain disulfides, also produces a heterogeneous mixture (typically DAR 0–8) but with a distribution centered around DAR 4. Another example is of trastuzumab emtansine, a widely known ADC, conjugated at lysine residue with a DAR from 0 to 8 having an average of 3.5 [23]. Site-specific conjugation strategies including engineered cysteine residues (THIOMABs) and unnatural amino acid incorporation, and enzymatic methods have been developed to produce homogeneous DAR2 or DAR4 conjugates with improved PK, narrower exposure–response relationships, and a wider therapeutic window [24]. Because individual DAR species differ measurably in hydrophobicity, plasma stability, and clearance, the overall conjugation strategy directly shapes the in vivo behavior of the molecule and is a central determinant of how the ADC must be parameterized in a PBPK model.

### 2.3. Key Determinants of In Vivo Fate

Once an ADC enters the systemic circulation, its fate is governed by a sequence of interlinked processes that together determine how much active payload reaches the tumor cell and what quantity of drug goes into healthy tissues. The main processes to be taken into account while developing a PBPK model are target binding at the tumor cell surface, receptor-mediated internalization, deconjugation in plasma, intracellular catabolism, FcRn-mediated recycling of the antibody, and the release and downstream fate of the catabolized payload [25]. The primary PK properties such as clearance, half-life, and tissue distribution of the ADCs are mainly governed by the antibody portion [26].

Target binding is the first determinant of selectivity. ADCs typically engage their tumor-associated antigen with high affinity (KD in the 0.1–10 nM range), and target abundance on the tumor cell surface (often 10^4^–10^6^ receptors/cell) sets the maximum theoretical payload that can be delivered. Heterogeneous expression across the tumor mass, together with the binding-site barrier, whereby high-affinity ADCs are sequestered by the first antigen-positive cells they encounter near the vasculature, limiting deep penetration, means that the effective tumor exposure can be substantially lower than the systemic exposure would suggest. After binding, receptor-mediated internalization channelizes the ADC-antigen complex into the endo-lysosomal system. The internalization rate constant (k_int_) varies considerably across targets and is itself a key efficacy determinant: rapidly internalizing antigens such as HER2 enabling efficient payload delivery, whereas slowly internalizing targets limit it.

Deconjugation occurs in parallel with these target-mediated processes. While the linker is designed to be stable in plasma, all linkers undergo some degree of non-specific cleavage in circulation: maleimide–thiol bonds are subject to retro-Michael exchange with serum albumin and free thiols, disulfide linkers are susceptible to reduction by glutathione and serum albumin, and peptide-based linkers can be cleaved by plasma carboxylesterases (notably mouse Ces1C, which can cleave Val-Cit linkers, and creates important species-specific differences in preclinical PK). Each deconjugation event reduces the DAR of an ADC molecule and releases free payload into the bloodstream, where it behaves like a small-molecule drug. Once internalized, the ADC undergoes intracellular catabolism: the antibody backbone is proteolytically degraded in lysosomes, and the linker is cleaved either by lysosomal acidity (acid-labile linkers), reductive conditions (disulfide linkers), or proteases such as cathepsin B (peptide linkers). Non-cleavable linkers do not release free payload at all; instead, the entire antibody is catabolized to release a charged amino acid-payload adduct that is largely retained intracellularly.

FcRn-mediated recycling acts as a counterweight to non-specific catabolism and is the basis for the long systemic half-life of the antibody scaffold. Antibody molecules taken non-specifically by endothelial and other cells via fluid-phase pinocytosis enter early endosomes, where the acidic pH (~6.0) drives pH-dependent binding of the Fc region to FcRn. The bound antibody is sorted into recycling vesicles and returned intact to the cell surface, where the near-neutral pH releases it back into circulation; the unbound antibody is trafficked to lysosomes for degradation. This salvage pathway is the reason an IgG1 has a plasma half-life of approximately 21 days rather than the few days expected from passive catabolism alone, and it is highly relevant to ADCs: the hydrophobicity introduced by payload conjugation, particularly at high DAR can disrupt the Fc-FcRn interaction, accelerating clearance of high-DAR species and skewing the in vivo DAR distribution towards lower values (Figure 2). Finally, once the payload is released, its downstream fate is governed by small-molecule disposition principles: efflux by transporters such as P-glycoprotein (P-GP), hepatic metabolism by Cytochrome P450 (CYP) enzymes (notably CYP3A4 for many auristatins), and biliary or renal excretion. Membrane-permeable catabolites can diffuse out of antigen-positive cells to reach neighboring antigen-negative cells, producing the bystander effect that broadens activity in heterogeneous tumors but also contributes to off-target toxicity. The impact of these factors while developing a PBPK model will be discussed in the upcoming sections. The dependence of ADC pharmacokinetic and physiological properties on linker chemistry is summarized in Table 2.

## 3. PBPK Model Architecture and Key Mechanistic Processes for ADCs

Physiological parameters describe the anatomy and physiology of the target organism. They are species-specific, measured independently of any ADC. They include organ volumes and tissue composition (determining interstitial and vascular volumes per tissue), cardiac output and tissue-specific blood flow rates, lymph flow rates, capillary pore sizes and densities for both small and large pore populations, and the endocytosis rate constant describing non-specific pinocytotic uptake by endothelial cells [41,42]. These values are species-specific and sourced from published physiological databases or the primary literature; they scale across species allometrically.

### 3.1. Model Hierarchy: Minimal, Semi-Mechanistic, and Whole-Body PBPK

The structural complexity of ADC PBPK models is determined by the scientific question, available data, and the stage of drug development. Minimal PBPK (mPBPK) models, represent plasma and a lumped peripheral tissue compartment, with protein transport governed by lymphatic flow and vascular permeability-surface area products calibrated for IgG-sized molecules. These models are well-suited for first-in-human dose prediction when tissue-level resolution is not required. In case of antibody-conjugated monomethyl auristatin E (acMMAE), a minimal PBPK model was developed using a three-compartment lumped model. Later, a single adjusting compartment (SAC) was used to better characterize the multi-compartmental plasma concentration profile of acMMAE [43]. Semi-mechanistic models introduce discrete organ compartments and DAR-explicit deconjugation, capturing the major drivers of nonlinear elimination without the full complexity of a whole-body [44]. Whole-body PBPK models includes each major organ divided into four standard sub-compartments (vascular, cellular, interstitial, and endothelial), incorporate the two-pore vascular extravasation model, include FcRn-mediated recycling within the endosomal system of each organ, and embed mechanistic payload sub-models providing the most complete representation of ADC PK but requiring the largest parameter sets [42].

### 3.2. Four Sub-Compartment Tissue Architecture

Unlike small-molecule drugs, which distribute across most tissue compartments by passive diffusion governed by lipophilicity and plasma protein binding, large-molecule ADCs (≈150 kDa) extravasate from the vascular space predominantly by convection through large paracellular pores and are actively recycled from the endosomal system by FcRn [45]. This becomes one of the core differences between the modeling of small-molecule drugs and the ADCs. The sub-compartment tissue architecture is a structural necessity for mechanistically describing the antibody component. If the endosomal compartment is not considered, the model becomes incapable of simulating FcRn-mediated half-life extension, the effects of FcRn engineering, or the impact of endogenous IgG competition on therapeutic ADC exposure. Omitting organ-specific interstitial sub-compartments hinders accurate prediction of tissue-to-plasma concentration ratios, which are critical for assessing both tumor penetration, which defines the efficacy, and off-target tissue exposure, which is responsible for defining the safety of the ADCs. In contrast, the payload once released behaves as a conventional small molecule and can be modeled using classical small-molecule PBPK principles creating a hybrid antibody–small-molecule coupled model as the complete ADC PBPK framework [42,46]. The four sub-compartments, the physiological basis for each, and the parameters required to specify them are summarized in Table 3.

#### 3.2.1. Vascular Compartment (Plasma and Red Blood Cell)

The first compartment encountered by any intravenously administered ADC is the vascular space, which is the capillary network within the organ. In humans, the total blood volume is approximately 5.5 L, of which roughly 55% is plasma. The heart pumps this plasma through the entire circulation at a cardiac output of around 5 L/min. Plasma contains albumin at 30–50 g/L and endogenous IgG at 8–18 g/L, both generating oncotic (colloidal osmotic) pressure that opposes protein filtration out of capillaries [41]. This oncotic balance is a primary determinant of the rate at which a therapeutic antibody extravasates into tissues and is a key input to the two-pore formalism described in the upcoming sections. For PBPK modeling of ADCs, the vascular compartment is divided into plasma and blood cells [47]. Within an organ, the ADC enters the vascular space (capillary compartment) through arterial perfusion (Q) and leaves via venous drainage, with the venous flow rate determined as the blood flow minus lymphatic flow (Q–L) [48].

#### 3.2.2. Endothelial Compartment

The endothelial cell layer constitutes the physical barrier between the blood and tissue interstitium. Its permeability varies substantially with capillary type: continuous capillaries (muscle, heart, lung) restrict protein passage; fenestrated capillaries (kidney, gut, endocrine glands) allow greater permeability; and sinusoidal capillaries in the liver, bone marrow, and spleen are essentially open to proteins of any size [49]. The blood–brain barrier represents the extreme of restrictiveness, with IgG concentrations in cerebrospinal fluid approximately 500-fold lower than in plasma [50]. These organ-specific permeability differences directly shape the ADC tissue distribution profile and must be parameterized organ-by-organ in a whole-body PBPK model. The endothelial compartment serves as a major site for the catabolic degradation of macromolecules following their uptake by fluid-phase endocytosis. However, Fc-containing molecules, including IgG-based monoclonal antibodies (mAbs) and albumin, interact with the FcRn, which protects them from lysosomal degradation and facilitates their recycling back to the circulation or transcytosis across tissues, thereby prolonging their systemic half-life [48].

#### 3.2.3. Interstitial Compartment

The interstitium is the fluid-filled space between cells, where the ADCs exert their pharmacological effect. In humans, the total interstitial fluid volume is approximately 12 L, of which roughly 6 L is accessible to proteins. Fluid enters by hydrostatic-pressure-driven filtration across the capillary wall and returns via lymphatic drainage at approximately 1.5 mL/min. Because proteins are partially excluded from the interstitial space, their concentrations are lower than in plasma: IgG lymph concentration is approximately 30% of plasma [41]. In addition to vascular distribution, antibodies may enter the interstitial compartment through lymph-driven convective transport and permeation across small and large endothelial pores, which is characterized by permeability–surface area processes [51]. The parenchymal cells present in this compartment, which uptakes the antibodies by cells via receptor-mediated endocytosis, in which internalization is triggered by their interaction with cell-surface receptors [48].

#### 3.2.4. Cellular Compartment

After internalization, the ADC–antigen complex is sorted along one of two pathways within the cell. One pathway is recycling back to the cell surface; another is trafficking to the lysosome for degradation [52]. For a trastuzumab–maytansinoid ADC, Maass and colleagues [53] quantified this processing kinetically, reporting half-lives of 6–14 h for internalization, 18–25 h for proteolytic degradation of the antibody backbone, and 44–73 h for efflux of the released payload, with internalization and efflux identified as the rate-limiting steps governing intracellular payload accumulation [53]. A comparable model developed for an epidermal growth factor receptor (EGFR)-targeted ADC found that up to 45% of internalized antibody could be recycled back to the cell surface rather than degraded, and that formation of free intracellular payload was delayed relative to antibody uptake, reflecting the additional time required for endo-lysosomal trafficking and subsequent proteolysis of the linker [54]. For cleavable linkers, payload is released within the endo-lysosomal compartment following proteolytic or acid-catalyzed cleavage; for non-cleavable linkers, release requires complete proteolytic degradation of the antibody backbone itself, yielding a charged amino-acid-payload catabolite that is largely membrane-impermeant. The cellular compartment therefore governs both the rate and completeness of payload delivery to its intracellular pharmacological target, and internalization and lysosomal processing rate constants are accordingly key inputs for cellular-level PBPK sub-models of ADC disposition.

### 3.3. FcRn-Mediated Recycling

PBPK models for ADCs have traditionally been descriptive in nature, often relying on in vivo tissue distribution data and focusing primarily on neonatal FcRn-mediated kinetics. These mechanistic models incorporate the major physiological process governing the disposition of the antibody, including fluid-phase pinocytosis, FcRn-mediated recycling, lysosomal degradation, and lymphatic transport. Following non-specific uptake into vascular endothelial cells through fluid-phase pinocytosis, the antibody component encounters an acidic environment (pH ≈ 6), where binding to FcRn directs it into recycling vesicles. Subsequent exocytosis and release at the cell surface under near-neutral pH (~7.4) return the antibody to the plasma or interstitial space, whereas unbound molecules are trafficked to lysosomes (pH 4–5) and degraded proteolytically [45]. This FcRn-mediated salvage pathway is responsible for the prolonged plasma half-life of IgG antibodies (~21 days) [55]. Modulation of the interaction between FcRn and IgG has been done by multiple macromolecular approaches. Among many approaches, mutating the Fc domain of the amino acid residue in proximity of the FcRn binding site supports lesser dosing frequency. Apart from increasing the half-life, FcRn modulation also results in improvement in the tissue distribution of the antibody as well as the drug carriers or associated alternative drug [55] in the ADC, as shown by nab-paclitaxel, an albumin-bound formulation of paclitaxel, where FcRn facilitated the distribution and penetration of nab-paclitaxel into target tissues while limiting its elimination into the intestinal lumen and subsequent fecal excretion [56].

Depending on the level of mechanistic detail required, PBPK models can be classified into minimal models (discussed in Section 3.1), which omit explicit endosomal compartments and are suitable for proteins with negligible FcRn interactions, and full PBPK models, which include endosomal recycling in individual organs and are particularly useful for evaluating Fc-engineered molecules and predicting antibody half-life [42,43].Advances in the in vitro characterization techniques and a growing understanding of ADC PK have enabled increasingly mechanistic PBPK frameworks that integrate in vitro and in vivo information to predict the disposition of ADCs and their components, analogous to approaches used for small molecules.

### 3.4. Target-Mediated Drug Disposition

Target-mediated drug disposition (TMDD) refers to a PK phenomenon where a drug binds with high affinity to its pharmacological target site such as a receptor or enzyme, to such a significant extent that this interaction impacts the drug’s distribution and elimination. The binding process often leads to nonlinear relationships between the dose and plasma concentration, particularly when the target concentration is comparable to the drug dose [44,57]. The prevalence of TMDD is common for large molecules, since these molecules have tendency to bind to their pharmacological target, and shows negligible non-target binding. The nonlinear relationship between the dose and plasma concentration is due to saturation of the binding target [57]. This nonlinear and dose-dependent PK is characterized by changes in the clearance and volume of distribution, due to elimination through target interaction. Furthermore, a decrease in the target during treatment may reduce target-mediated clearance upon repeated dosing, resulting in increased systemic exposure over time. Importantly, TMDD can also contribute to off-target toxicity when the target antigen is expressed in healthy tissues [58]. For example, studies with anti-MET ADCs demonstrated that reducing antibody affinity diminished TMDD in normal tissues while preserving tumor uptake, thereby improving the therapeutic index [59]. Collectively, these findings highlight TMDD as a critical pharmacokinetic–pharmacodynamic determinant governing both the efficacy and safety of ADCs.

### 3.5. Two-Pore Formalism and Vascular Extravasation

The two-pore hypothesis, originally formulated by Rippe and Haraldsson) [60], postulates that the transcapillary passage of macromolecules occurs through two populations of paracellular pores: numerous small pores (radius ~4–5 nm) and a sparse population of large pores (radius ~20–30 nm). For a 150 kDa monoclonal antibody with a hydrodynamic radius of approximately 9 nm, the molecule is too large for free passage through small pores and relies predominantly (~90%) on bulk convective flow through large pores for extravasation. By contrast, a 12 kDa nanobody (~3 nm radius) is transported predominantly (~90%) by diffusion through small pores [42]. The hydrodynamic radius derived from molecular weight via a power-law relationship as defined in equation 1 below. It is a key size parameter linking ADC chemistry to its transport behavior.

*Rh* = *a* × (*MW*)*^b^* (1)

where *Rh* = hydrodynamic radius;

*MW* = molecular weight;

*a* and *b* = empirical constants for proteins (differ for each type of proteins).

By specifying small-pore and large-pore radii for each organ, the model predicts size-dependent reflection coefficients, convection and diffusion coefficients, and permeability-surface area products, generating organ-specific interstitial-to-plasma concentration ratios consistent with radiolabeled antibody tissue distribution data [46].

## 4. PBPK Model Development for ADCs

The ADME properties of ADCs differ significantly from their drug payload [61]. For the development of a full-fledged PBPK model for an ADC, a sequence of data is required. Initially, the biodistribution data for the mAb and the drug payload in any species is required. This includes experimental data about, but not limited to, the percent of dose present in each organ after dose administration. To develop the PBPK model, several key monoclonal antibody (mAb) disposition characteristics can be incorporated based on their availability. These include systemic circulation through organs and tissues, transcapillary transport modeled via the two-pore theory, and lymphatic return from the interstitial space back into blood circulation. Additionally, the model accounts for the specific binding of the antibody to tumor-associated antigens, its interaction with FcRn receptors in the endothelium of the liver, skin, and muscle, and its overall catabolic clearance, concluding with the renal elimination of the resulting catabolic waste products.

### 4.1. Model Structure and Physiological Representation

The required organs and tissues for developing the model include one lumped compartment. Since the antibody component of an ADC is a large molecule, its disposition is described using a dispersion tissue model in which each organ is divided into distinct sub-compartments. Transport of both the large and small molecule across the capillary endothelium in each organ is represented using a two-pore model, with passive diffusion and convection as the primary mechanisms governing macromolecular transfer. In the case of modeling the ADC containing MMAE as the cytotoxic payload and IgG as the mAb, Chang and Shah demonstrated two model structures for each component, where they were connected through deconjugation and degradation process, a generic organ level representation (Figure 3). The connection between the compartments occurs by blood and lymphatic flow [47,62]. The sub-compartment architecture within each tissue is detailed separately in Figure 4 in below section on Two-Pore Theory, FcRn-Mediated Recycling and Endosomal Trafficking.

#### Two-Pore Theory, FcRn-Mediated Recycling and Endosomal Trafficking

In the two-pore framework, fluid and macromolecules traverse the vascular wall via large pores, whereas fluid and small solutes are transported through small pores. Passive diffusion and convection are the two mechanisms used to describe the transport of the large molecule across the capillary endothelium [63]. This framework allows the model to simultaneously capture the restricted transvascular exchange of the antibody and the comparatively unrestricted movement of the liberated small-molecule payload once it is released intracellularly.

To capture FcRn-mediated recycling, an intracellular endosomal sub-compartment is commonly incorporated into ADC PBPK models. Following transvascular distribution, ADCs or their antibody components are internalized by endothelial cells through fluid-phase pinocytosis. Within the acidic endosomal environment, Fc-containing molecules bind to the FcRn, which protects them from lysosomal degradation and facilitates their recycling back to the plasma or interstitial space, thereby contributing to their prolonged systemic half-life. In contrast, unbound molecules are directed to lysosomes for catabolism, with degradation products subsequently cleared from the tissue. Tissue-specific expression of FcRn is therefore an important determinant of ADC disposition and persistence in vivo. This endosomal sub-compartment is incorporated into the liver, skin, and muscle in the model to reflect the known sites of FcRn expression.

When building ADC PBPK models, key assumptions often include DAR-dependent payload release following intracellular degradation of the conjugate, first-order deconjugation in systemic and tissue compartments, and similar distribution and FcRn-mediated recycling properties for the ADC and the corresponding unconjugated antibody. Released payload species are typically modeled according to their intrinsic small-molecule PK characteristics. In addition, non-specific deconjugation processes can be incorporated across plasma, interstitial, and intracellular spaces to capture payload release occurring outside target cells. These assumptions provide a mechanistic framework for describing the disposition of intact ADC, total antibody, and released drug payload species [62].

A detailed system of differential equations describing the PK of the released drug payload in plasma and tissue sub-compartments was thoroughly developed by Chang [47]. Their model incorporates organ-specific vascular, blood cell, interstitial, endosomal, and cellular spaces, together with physiological blood flows and permeability parameters, enabling a mechanistic description of drug payload disposition (Figure 4). The model equations account for tissue-specific blood flows, compartmental volumes, permeability coefficients, and intracellular trafficking processes, providing a detailed representation of payload distribution and elimination. Interested readers are referred to the original publication for the complete mathematical formulation and parameter definitions [47].

### 4.2. Parameterization of ADC PBPK Models

Parameterization of ADC PBPK models requires the integration of both physiological and drug-specific parameters governing the distribution, elimination, and target interaction of the conjugate. These parameters are derived from a combination of experimental measurements, literature values, in vitro studies, in silico predictions, and parameter estimation through model fitting. In situations where experimental data are unavailable, assumptions and scaling approaches are often employed, highlighting the importance of sensitivity analyses and model validation in establishing confidence in ADC PBPK predictions.

#### 4.2.1. Physiological Parameters

Physiological parameters are system-level inputs that are largely species-specific and independent of the drug candidate. They are typically sourced from published physiology databases or the literature and include organ blood flow rates (Q), transcapillary fluid filtration rates (J), lymphatic flow rates (L), vascular, interstitial, and cellular volumes per organ, permeability–surface area products (PS), rates of intracellular catabolism (k_aat_), clearance of degradation products (CL) and urinary excretion rates, tissue-specific FcRn internalization rate constant (k_int_) and recycling rate constant (k_rec_).

Additional parameters describing target-mediated processes include antigen expression levels, target density per cell, and antigen turnover rates. These are drug–target combination-specific and must be established for each ADC.

#### 4.2.2. Antibody- and ADC-Specific Parameters

The antibody-related parameters include the type of mAb, its molecular size, tumor antigen concentration, osmotic reflection coefficient, specific binding rate constants, and permeability–surface area products [63].

Endosomal parameters govern the FcRn recycling cycle and determine the extended plasma half-life characteristic of IgG-class antibodies and ADCs. Key endosomal parameters include the FcRn dissociation constant (Kᴅ) at pH 6.0 (governing endosomal binding) and at pH 7.4 (governing release at the neutral cell surface), endosomal compartment volumes, recycling fraction, endosomal recycling and degradation rate constants, the FcRn tissue concentration and the endogenous IgG synthesis rate, which set the competition baseline for FcRn binding [45,64].

ADC-specific physicochemical and PK parameters vary between individual drug candidates and must be measured or estimated for each molecule. These include the molecular weight, from which the hydrodynamic radius is derived via a power-law relationship, the primary size descriptor governing two-pore transport, the net charge (negatively charged proteins undergo more efficient glomerular clearance) [65], blood-to-plasma concentration ratio, unbound fraction in plasma (fᵤ), osmotic reflection coefficients (σ) for large and small pores, and tissue partition coefficients for the liberated payload.

For TMDD-relevant ADCs, target-binding kinetic parameters are also required. Parameters like the drug–target association rate (k_on_), dissociation rate (k_off_), and complex internalization rate (k_int_) are typically derived from in vitro cell-binding assays. Deconjugation rate constants for each DAR species are determined from plasma stability experiments or by fitting preclinical PK data. Linker hydrolysis is governed by linker chemistry rather than species biology and is therefore transferable across species. Payload permeability and tissue binding constants are molecular constants directly transferable from in vitro measurements to the human PBPK model once the small molecule is liberated [66].

#### 4.2.3. In Vitro-to-In Vivo Extrapolation

The measurement of plasma concentrations of intact ADC, total antibody, and free payload often correlate poorly with target-site (tumor) exposure, and direct sampling of human tumor tissue is neither ethical nor feasible. Hence, effective parameterization of ADC PBPK models requires a systematic approach to in vitro-to-in vivo extrapolation (IVIVE), translating in vitro assay results to in vivo model inputs with appropriate scaling factors. Hepatic clearance of the payload is scaled from microsomal incubation data using intrinsic hepatic clearance models with liver blood flow and unbound fraction corrections. Tissue binding parameters for the payload are estimated from equilibrium dialysis assays in relevant tissues [67]. In vitro target-binding kinetics (k_on_, k_off_) and receptor internalization rate (k_int_) if not found in the literature are typically measured by cell-based binding assays and can be integrated directly into mechanistic tumor disposition models to generate a priori predictions of tumor mAb, ADC, and released-payload concentrations ahead of in vivo data. In a study, this approach was demonstrated for an auristatin-based anti-5T4 ADC, where the sensitivity analysis further identified payload dissociation rate and tumor size as critical determinants of payload exposure in both plasma and tumor tissue [68]. DAR-dependent deconjugation rates, if not directly measurable for all species, can be derived from fitting a DAR-explicit model to total antibody and intact ADC plasma time-courses from preclinical studies [69]. Deconjugation rate constants were measured directly by incubating the ADC in vitro species-relevant plasma at 37 °C and monitoring the decline in total antibody and individual DAR moieties by ELISA/LC-MS, fitting the resulting concentration-time data to first-order kinetics, to mechanistically distinguish the chemical instability of the linker from the biological clearance of the antibody backbone for trastuzumab emtansine [70].

## 5. Applications of PBPK Modeling in ADC Development

PBPK models play a crucial role in the development of ADCs. This section discusses a few important applications. ADCs carry a potent cytotoxic payload that can cause serious off-target tissue damage if released from the antibody scaffold. The primary driver of ADC toxicity is free payload in systemic circulation arising from non-specific linker deconjugation: peripheral neuropathy from tubulin-targeting payloads (DM1, MMAE), hepatotoxicity from calicheamicin, and hematological toxicity from topoisomerase inhibitors (SN-38). Toxicokinetic (TK) and pharmacodynamic–toxicodynamic (PD/TD) models for ADCs link free payload concentrations in relevant off-target tissues to toxicity endpoints using exposure-response models [71]. PBPK models are essential for defining the maximum tolerated dose and informing dosing schedule optimization to maximize the therapeutic window.

One important application of PBPK modeling in ADCs is the estimation of the systemic exposure of multiple analytes. PBPK predicts plasma profiles of intact ADC, total antibody, conjugated and unconjugated payload as distinct species, something conventional PK cannot resolve. An ADC molecule exists in a dynamic state after administration, i.e., the payload undergoes deconjugation and FcRn binding. PBPK modeling overcomes the limitations of conventional PK by linking monoclonal antibody distribution, endosomal processing, and FcRn recycling directly to a small-molecule sub-model which incorporates payload-specific parameters like hepatic clearance, renal elimination, and membrane permeability. These processes mentioned above are occurring parallelly, and PBPK models help to capture the biological reality by incorporating the said processes in one place.

Another significant application of this approach is the prediction of DDI and the effect of organ impairment. For instance, upifitamab rilsodotin is an ADC carrying the auristatin F-hydroxypropylamide as drug payload. A PBPK model was built using in vitro, animal and clinical data to predict DDI and the effect of hepatic impairment. Both a top-down and bottom-up approach were used to develop the model. The payload is metabolized by CYP3A4 and is also a P-glycoprotein substrate; so, an interaction was plausible. The model predicted only a small effect of about a 30% rise in unconjugated drug payload exposure when given with itraconazole and almost no change in patients with mild liver impairment, which matched the clinical data. A larger rise of about 1.5-fold in the AUC of unconjugated drug payload and a negligible change in C_max_ was predicted for moderate and severe hepatic impairment. The same model can be adapted for other ADCs that share the linker [72]. A similar approach was taken for telisotuzumab vedotin with MMAE as drug payload, where simulations replaced a clinical DDI study and supported the advice to monitor patients who take strong CYP3A inhibitors or inducers as comedication [73].

As ADCs are multi-analyte molecules, simple empirical allometric scaling is inadequate for IVIVE. PBPK modeling provides a mechanistic framework for IVIVE and cross-species translation of ADC PK from preclinical species to humans, supporting first-in-human dose selection and clinical trial design. PBPK modeling has two independent categories: physiological parameters that change across species (organ volumes, blood flows, lymph flow, FcRn expression, endogenous IgG levels, target abundance), and drug-specific molecular parameters that remain constant across species (FcRn binding affinity, target binding/internalization kinetics, deconjugation rate, payload permeability), which are measured once in vitro and carried forward unchanged. This means that when a human prediction is inaccurate, errors can be traced to a specific physiological input [46,69]. Cynomolgus monkeys are preferred for this scaling step because of their FcRn sequence homology, their antigen expression patterns on normal tissue resemble humans, and an IgG1 half-life (~14–18 days) far closer to the human value (~21 days) than mouse (~6–8 days) [74,75]. In a recent study, PBPK modeling was used to reduce the reliance of animal testing for guiding first-in-human doses. Benamara and colleagues investigated cross-species extrapolation for large molecules by estimating the neonatal Fc receptor dissociation constant (K_d_) across 27 antibodies in humans, monkeys, rats, and mice using a generic PBPK model. Because in vitro assays vary, and animal-to-human regressions showed weak correlations, direct extrapolation proved unreliable. However, when a two-fold error was tolerated, both rat-based extrapolation and a fixed median human value of 1.52 µM successfully predicted over 90% of human K_d_ values, demonstrating that a default human value is a viable alternative when animal data are unavailable [76]. Building on this, Benamara and colleagues expanded the approach using a clinical database of 50 intravenous monoclonal antibodies, randomly split into training and test sets. Using the generic large-molecule model in PK-Sim, they estimated K_d_ as the sole fitted parameter. The authors then performed naive predictions, i.e., predicting human PK profiles using a default parameter value without any additional drug-specific data fitting. Applying the validated overall median K_d_ value of 1.07 µM, these naive predictions accurately estimated plasma exposure in terms of AUC and terminal half-life for 80% and 60% of the drugs in the test, respectively. For these compounds, the prediction error was in the 0.80–1.25-fold range. These studies show that PBPK modeling offers a viable alternative and helps in reducing reliance on animal studies, although a thing to be noted here is that these predictions are only applicable for compounds with linear PK [77]. To provide novel and efficient dose selection with minimal preclinical data, allometric scaling was applied to the PK parameters of monkeys for the prediction of clearance and volume of distribution in humans. The predicted human payload exposures showed good agreement with clinical observations, with most predictions falling within three-fold of the observed values across different types of linkers [75].

Risk assessment and safety margin evaluation is another important application of the PBPK models in ADC development. While the main purpose of introducing ADC for cancer treatment is the reduction in drug related toxicity, the toxicity caused due to the bystander effect cannot be discounted. In the bystander effect, the drug payload often gets diffused to the neighboring cells from the target cells [78]. This can be even more prominent in non-polar payloads due to their high membrane permeability. Common toxicity includes hematological, gastrointestinal, peripheral, ocular, dermal, embryo–fetal toxicity, and interstitial lung disease [79]. From the toxicokinetic point of view, the drug payload is considered to be the primary cause of the toxicity occurring due to ADCs. This has resulted in the discontinuation of several ADC formulations. In several cases, non-specific targeting apart from the bystander effect also caused toxicity. For instance, MMAE is a payload, which is part of three approved ADCs (Table 1). Among these, brentuximab vedotin and enfortumab vedotin show adverse-effects like peripheral neuropathy, neutropenia, and alopecia. These recurrently observed adverse events that correlate with plasma-free MMAE exposure rather than intact ADC exposure [80,81]. PBPK modeling provides a framework for the ADC bystander effect, since it links whole-body disposition to local tumor-tissue processes governing payload spread. To model the bystander effect, the tumor compartment is split into antigen-positive (Ag+) and antigen-negative (Ag–) subpopulations, as the payload diffuses out of targeted cells to kill neighboring cells that lack the target antigen [13]. Khera and colleagues added a transport sub-model linking payload distribution to antibody/payload dose and diffusivity [82], while Byun and colleagues used age-structured kinetics to capture bystander killing [83]. It is evident that off-target payload distribution is one of the major reasons for ADC toxicity. A PBPK-PD model was built for trastuzumab deruxtecan and sacituzumab govitecan, first in tumor-bearing mice and then scaled to breast cancer patients. For 1000 virtual patients, the predicted AUC and C_max_ of ADCs and the release drug payload fell within two-fold of the measured values, and the predicted progression-free survival matched the clinical observation. The model also linked payload concentration in the lung to the risk of interstitial lung disease and showed that this risk rose with age and higher dose. A sensitivity analysis found that the antigen expression level and the rate at which the antigen–ADC complex is internalized were the main factors determining the distribution of ADC. This is useful because both can be measured before a trial begins [84]. Together, these studies illustrate how PBPK modeling provides a robust platform for predicting bystander-driven efficacy and guiding ADC dose and design decisions.

### Critical Analysis of the Reported Application

Most of these successful applications have their own limitations. For instance, in the DDI and effect of organ impairment, predictions deal only with the drug payload after it is released from the ADCs. Hence, it treats the drug payload as the conventional small molecule [72,73]. This gives information about the drug payload; however, the information about the antibody is not considered in such cases. The PBPK-PD model for trastuzumab deruxtecan and sacituzumab govitecan was verified against plasma concentrations and progression-free survival, yet its conclusion about interstitial lung disease depends on the payload concentration in the lung, which was predicted and not measured [84]. A detailed discussion about this is mentioned in the upcoming Limitations Section (Section 7). In the cross-species extrapolation studies, the FcRn studies were carried out on unconjugated monoclonal antibodies; so, they strengthen the antibody sub-model but tell nothing about the deconjugated payload or payload disposition [76,77]. In another example of cross-species extrapolation, where clearance and volume of distribution is estimated using the PK parameter of monkeys, this predicts the parameters within two- to three-fold, which is sufficient for selecting a starting dose but not for judging a therapeutic index [75]. Experimental data fed into the model as an input parameter adds another constraint to the model. For instance, in case of ligand-binding assays, individual DAR species cannot be separated. In addition, they poorly detect unconjugated or partly conjugated payload [61]. Variation between laboratories in how inputs are measured can also produce large prediction errors, which is why sensitivity analysis and parameter tuning based on the observed data is used as a corrective strategy [85]. Hence, it can be said that the quality of an ADC PBPK model depends on the analytical methods carried out to derive the input parameters and on the structure of the model itself.

## 6. Regulatory Landscape for PBPK Modeling of ADCs

The USFDA in its Guidance for Industry on Clinical Pharmacology Considerations for Antibody–Drug Conjugates states that ADCs are regulated as biological products and are therefore subject to applicable laws and regulatory requirements governing their development, evaluation, and approval under Section 351 of the Public Health Service (PHS) Act (42 U.S.C. 262). The guidance identifies drug–drug interaction (DDI) as an important consideration in ADC development and recommends an initial in vitro assessment of DDI risk with in vivo DDI characterization considered where appropriate. It further states that PBPK modeling could be appropriate for DDI assessment. PBPK analyses can complement in vitro and in vivo ADC component evaluations and support the characterization and risk assessment of interactions involving ADC drug payloads and relevant constituent parts of the ADC [86]. ADCs come under the biologics license act (BLA), according to the guidance for industry on physiologically based PK analyses—Format and Content document [87]. According to this guidance, it is suggested that sponsors should submit enough information to the FDA, so that the reviewers should be able to simulate and replicate the model and conduct any supplementary analysis if needed. It is suggested that PBPK reports should provide a clear description of the modeling strategy, model parameters and assumptions, simulation design, software and supporting electronic files, as well as comprehensive documentation of model verification, modification, and application. The FDA recommends presenting parameter sources, sensitivity analyses, and simulation results using appropriate tables and graphical summaries to demonstrate model robustness and suitability for the intended scientific, clinical, or regulatory purpose. The FDA recommends that sponsors must clearly demonstrate how PBPK modeling and simulation analyses address the relevant scientific, regulatory, and clinical questions. PBPK models may be used to support waivers of clinical studies, characterize DDI risks, and inform dosing strategies for ADCs. Any dosing recommendations derived from PBPK simulations should be justified within the overall body of evidence, including established exposure–response relationships and the level of confidence in the model for its intended application. Such analyses may ultimately support dosage recommendations incorporated into product labeling [87].

In the context of the use of PBPK for deducing DDI in case of ADCs, a PBPK model-based strategy was developed by different researchers by adapting existing small-molecule DDI frameworks for biologics and incorporating clinical learnings from other ADCs to evaluate the CYP3A-mediated DDI potential of polatuzumab vedotin, an anti-CD79b vc–MMAE ADC. The approach was cross-validated using clinical DDI data from brentuximab vedotin, a structurally similar vcMMAE ADC. Consistent with brentuximab vedotin, PBPK simulations at the proposed clinical dose suggested that polatuzumab vedotin is unlikely to exhibit clinically meaningful interactions with strong CYP3A inhibitors or inducers and is not expected to meaningfully inhibit or induce CYP3A [43]. This work illustrates an application of model-informed drug development (MIDD), supporting Biologics License Application (BLA) and Marketing Authorization Application submissions to predict CYP3A-mediated DDIs and inform labeling in the absence of dedicated clinical DDI studies [88,89,90,91].

## 7. Limitations and Current Challenges of PBPK Modeling for ADCs

The earlier sections described what PBPK modeling can currently deliver for ADCs’ PK prediction. However, it is equally important to highlight the limitations of this approach, because the mechanistic appearance of a PBPK model can convey more confidence than the underlying data support. The constraints outlined below are applicable for every published ADC PBPK model and should be taken into consideration when such models are used to support development or regulatory decisions.

The major limitation of a PBPK model of ADCs is that it is calibrated mostly against plasma concentrations of intact ADC, total antibody and free payload, whereas the quantity that determines the efficacy is the payload exposure within the tumor. As noted in Section 4.2.3, serial sampling of human tumor tissue is neither ethical nor practical; so, predicted tumor concentrations remain unverified in patients and are extrapolations rather than validated outputs. The models are also limited by heterogeneity in antigen expression [92] and tumor microdistribution, which may not be adequately captured by tissue-level compartments [85]. In addition, ADC structural heterogeneity, particularly differences in DAR and time-dependent deconjugation, can influence distribution and clearance but are often simplified. The models are subject to substantial parameter uncertainty and intertumoral/interpatient variability, as well as challenges in extrapolating model parameters from preclinical species to humans [5].

While physiologically based pharmacokinetic (PBPK) models are instrumental in predicting the clinical disposition of ADCs, structural simplifications regarding payload release kinetics can introduce uncertainties into therapeutic index estimations. For example, a recent model for telisotuzumab vedotin optimized deconjugation only for the DAR 1 species, and rates for higher-order DAR species were extrapolated using a fixed default. The model assumed that that deconjugation proceeds at an identical rate in both systemic plasma and peripheral tissues. Systemic payload drives off-target toxicity, and local payload dictates tumor efficacy; mathematically locking these rates together artificially predefines their balance. This constraint results in a deceptive goodness-of-fit for systemic plasma exposures while entirely masking the true unmeasured variability of local tumor pharmacokinetics [73]. Another additional limitation is that important systemic clearance pathways may not yet be mechanistically represented in PBPK models. A recently published analysis of biologic PBPK models, including ADCs, found that simulations systematically overpredicted exposure when only established clearance pathways such as lysosomal catabolism and renal filtration were considered. This necessitates an additional systemic clearance pathway to improve prediction. However, this additional clearance pathway currently requires fitting to observed data, limiting its use for de novo predictions. The authors further noted that potential clearance mechanisms, including uptake by antigen-presenting cells, Fcγ receptor-mediated uptake, pinocytosis by other cell types, and hepatic uptake, are not yet sufficiently characterized to be incorporated mechanistically. Increasing the number of mechanistic clearance pathways may also introduce parameter-identifiability problems when only plasma concentration data are available. Thus, although adding an empirical clearance can improve model performance, it does not necessarily resolve the underlying mechanistic uncertainty and may limit the predictive capability of ADC PBPK models for new molecules or clinical settings [93].

Hence, it can be said that a good plasma fit in the case of simulated data of ADCs does not necessarily mean that the unmeasured mechanistic processes governing efficacy and toxicity are correctly represented.

## 8. Future Directions

In the current scenario, among the 245 FDA-approved new drugs during year 2020–2024, where a PBPK model has been provided as an important evidence, New Drug Application (NDA) and Biologics License Application (BLA) have a share of 26.5% [94]. This shows that PBPK is playing an important role during the regulatory submission of biological drugs including ADC. The role of IVIVE is very well known in PBPK modeling, where the in vitro generated data can be used to either parameterize the model or support risk assessment with the capability for becoming NAM [95]. NAMs encompass emerging in vitro, in silico, and other advanced testing platforms aimed at evaluating the safety and efficacy of drugs and biologics. By providing more human-relevant assessments of toxicity, immunogenicity, and pharmacodynamics, NAMs have the potential to enhance the predictive value of nonclinical studies while reducing or replacing animal use [96]. Combining PBPK models with multi-omics approaches has the potential to markedly enhance predictive capabilities, thereby providing actionable information for precision medicine applications including ADCs. In addition to this, artificial intelligence (AI) has the potential to increase the accuracy of PBPK models and harmonize model validation standards. Recent advances have demonstrated the integration of AI/ML models with PBPK models to enable the rapid prediction of drug PK from molecular structure [97]. This becomes even more impactful for heterogenous modalities like ADC due to the dynamic nature of the average number of drug payload attached to each antibody [98,99]. For example, researchers have successfully upgraded standard PBPK models, such as the widely used Shah and Betts model, using AI to better understand how drug clearance varies between different molecules. Tomasoni and colleagues applied this idea to Fc-fusion proteins. Using 41 de novo designed Fc-fusion proteins with plasma PK profiles measured in mice after a 2 mg/kg intravenous dose, they computed 55 physicochemical properties in silico for each protein from AlphaFold-predicted structures. Because many of these properties were strongly correlated, they were grouped by hierarchical clustering and reduced to 15 representative descriptors. The authors then modified the Shah and Betts model by introducing two protein-specific scaling factors, F1 and F2, which adjust the non-specific cellular uptake rate (CL_UP_) and the lymph-to-blood return rate (CL_LNLF_), respectively. Using a bounded symbolic regression procedure, which combined random sampling and genetic optimization and was guided by a performance score that favors simpler formulas, they derived simple analytical formulas linking the logarithms of F1 and F2 to a small set of structural properties, including the apparent charge, the isoelectric point of the Fc-fusion protein, positively charged surface patches, and hydrophobic patches. In this way, the model captures how electrostatic and hydrophobic characteristics of a molecule influence its uptake by cells and its recycling from tissues back into the blood. The modified model was fitted on 34 proteins, validated on 7 independent proteins and showed markedly lower prediction errors than the original Shah and Betts model [100].

ML and AI techniques offer major advantages by including the ability to identify strategies for reducing the parameter space, i.e., the massive collection of all possible variables, molecular features, and mathematical combinations a model must evaluate. In complex drugs like ADCs, trying to calculate every possible biological and chemical interaction creates an overwhelmingly large “space” that can make mathematical models slow, inaccurate, and unstable. To make this manageable, AI tools groups similar features together to shrink the number of variables, preventing the model from getting confused by too much overlapping data. They can also enhance confidence in the estimated values of the parameters that have the strongest influence on model outcomes. It is achieved by running sensitivity analyses to pinpoint exactly which variables drive the model’s behavior. Once these critical factors are identified, algorithms repeatedly sample different combinations of values to converge on a solution, fine-tuning the model step-by-step to minimize prediction errors. This ultimately improves the prediction reliability [100,101]. There are a limited number of studies for the application of AI tools or ML models in PBPK modeling, and this is a topic that can be explored in the future.

## 9. Conclusions

ADCs present unique PK challenges arising from their multi-analyte nature, intact conjugate, total antibody, and free payload, each governed by distinct biological processes within a narrow therapeutic window. Conventional PK approaches cannot adequately resolve this complexity, making PBPK modeling an essential tool for ADC development. The mechanistic framework described in this review, incorporating multi-compartment tissue architecture, two-pore transvascular transport, FcRn-mediated recycling, TMDD, and a coupled small-molecule payload sub-model, enables the simultaneous prediction of ADC disposition across plasma, tumor, and other off-target tissues. The clear separation of species-specific physiological parameters from invariant drug-specific molecular constants makes this framework particularly powerful for cross-species translation and first-in-human dose projection.

Beyond dose prediction, PBPK models have demonstrated regulatory utility in characterizing DDI risk and informing product labeling without dedicated clinical studies, as accepted by both the FDA and EMA. Integration with toxicodynamic and tumor-response models represent the logical next step, enabling fully coupled exposure–response simulations to support adaptive trial design. PBPK modeling will remain the important aspect of the development of any new drug delivery system including ADCs as there are many ADCs in the research and development pipeline.

In this review, we examined PBPK models that separately track intact ADC, total antibody, and free payload and found that they predict exposure better than conventional PK approaches. Tumor exposure remains less well established, because current models are validated mainly against plasma data rather than tumor data. Human tumor tissue is difficult to sample, antigen expression varies within tumors in ways that models do not fully capture, and DAR-dependent deconjugation is often simplified; so, tumor-level predictions carry meaningful uncertainty. Overall, PBPK modeling is a powerful and increasingly reliable framework for ADC development, and integrating NAM and AI approaches should strengthen it further.

## Figures and Tables

**Figure 1:**
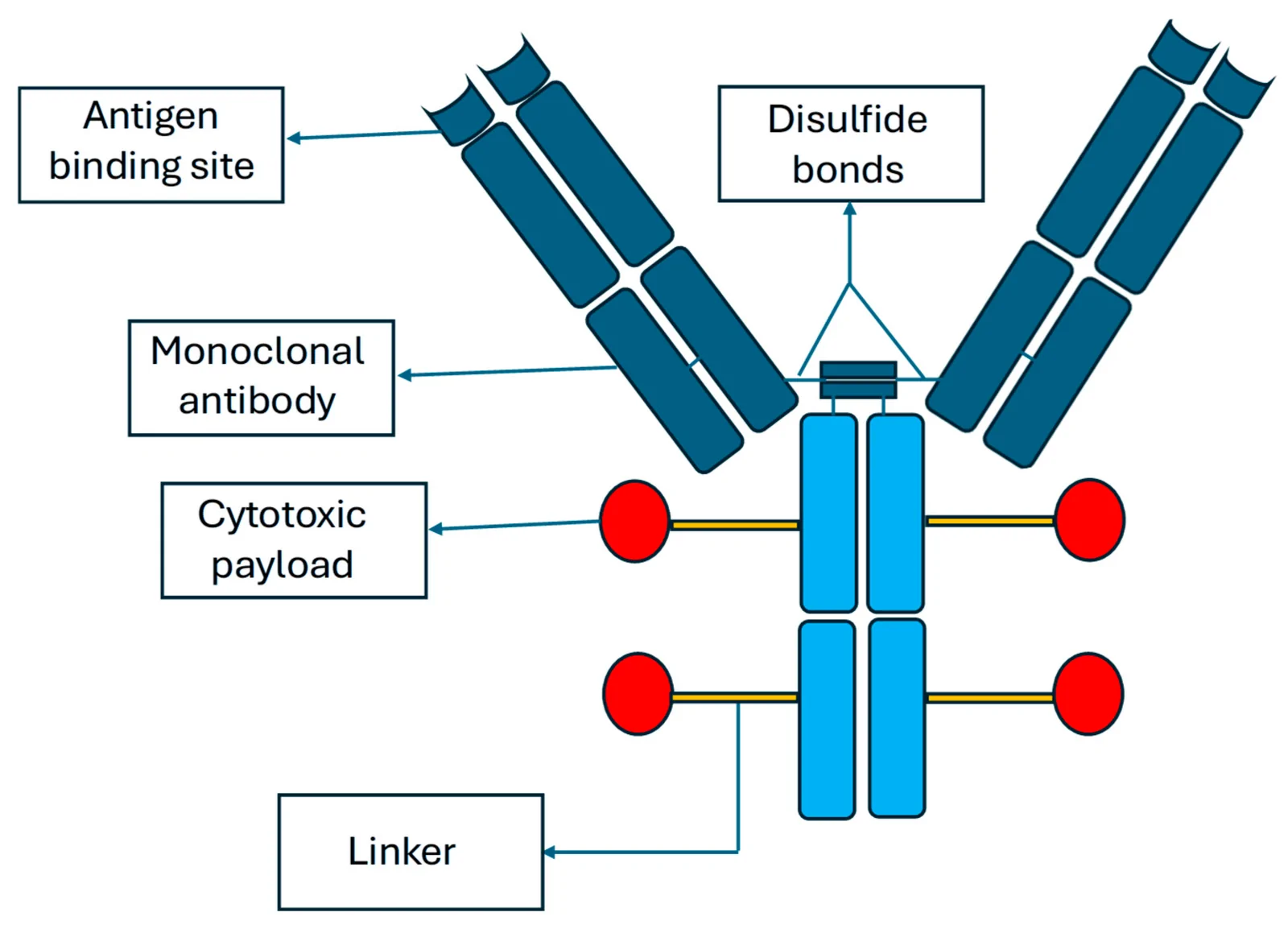
Structure of a typical antibody–drug conjugate. The monoclonal antibody confers selectivity for a tumor-associated antigen, the chemical linker determines the site and rate at which the payload is released, and the cytotoxic payload produces the pharmacological effect. The number of payload molecules per antibody defines the drug-to-antibody ratio (DAR).

**Figure 2:**
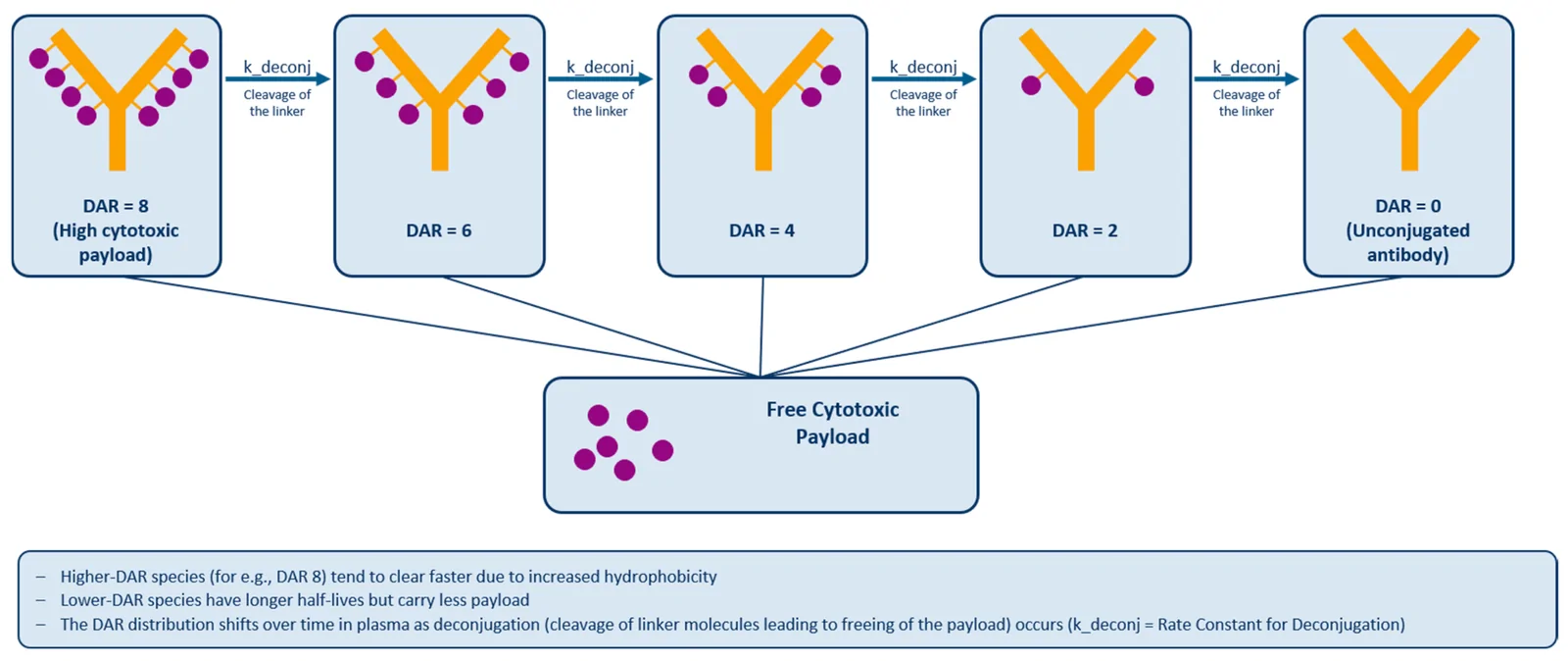
Stepwise deconjugation of the ADC and its effect on plasma DAR distribution. Linker cleavage (k_deconj) sequentially converts DAR 8 to DAR 0 species, releasing free cytotoxic payload at each step; higher-DAR species clear faster due to increased hydrophobicity, skewing the in vivo DAR distribution toward lower-DAR species over time. DAR: drug-to-antibody ratio.

**Figure 3:**
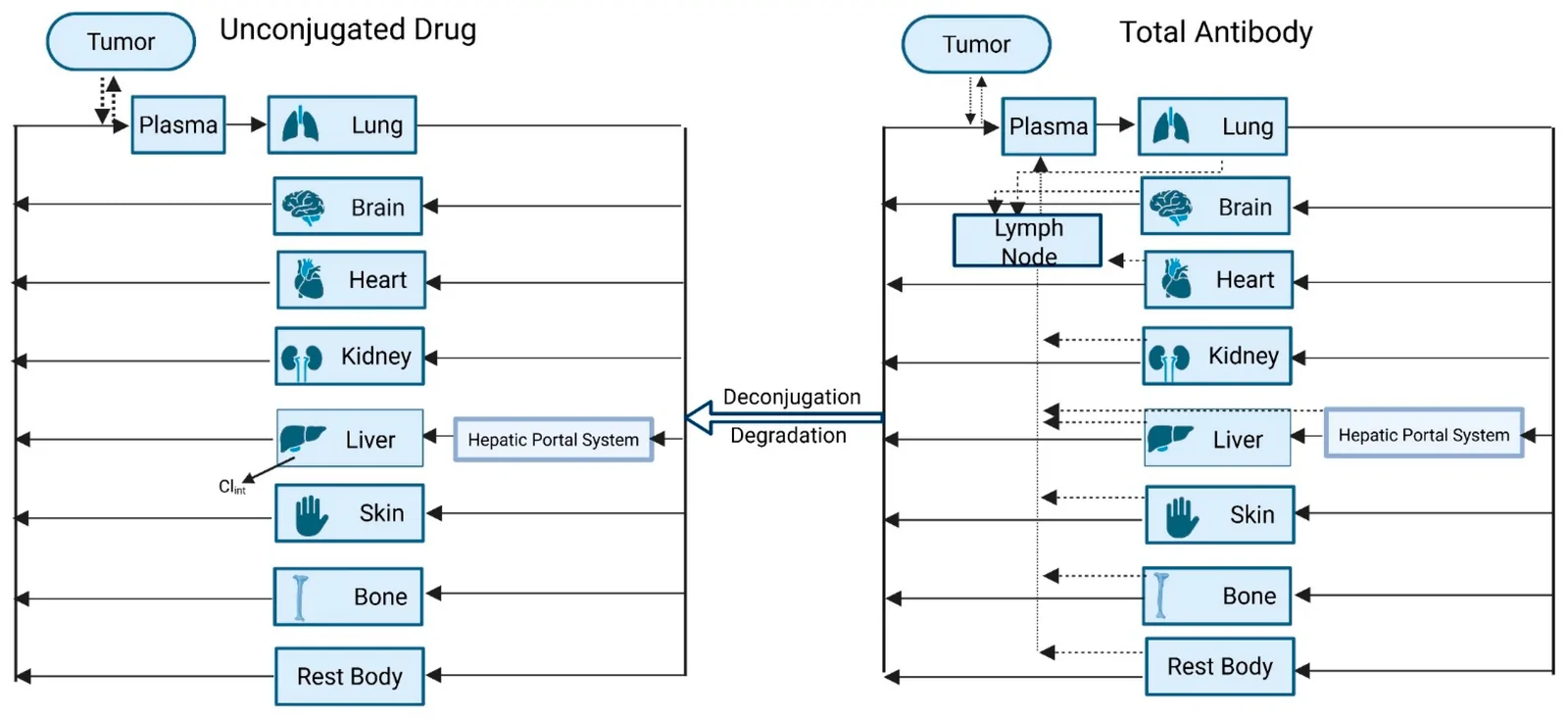
Generic representation of whole-body (organ level) PBPK model structure for the disposition of ADC analytes, showing the dual-model architecture used to describe (**left**) the unconjugated small-molecule payload and (**right**) the total antibody component. The two sub-models are mechanistically linked through deconjugation and degradation processes, representing tissue-level release of unconjugated payload from the antibody–drug conjugate. Solid arrows denote plasma/blood flow; dashed arrows denote lymphatic flow, present only in the total antibody model via an additional lymph node compartment accounting for convective lymphatic return of the antibody to systemic circulation. Model structure adapted from the whole-body PBPK framework described by Chang and Shah [51].

**Figure 4:**
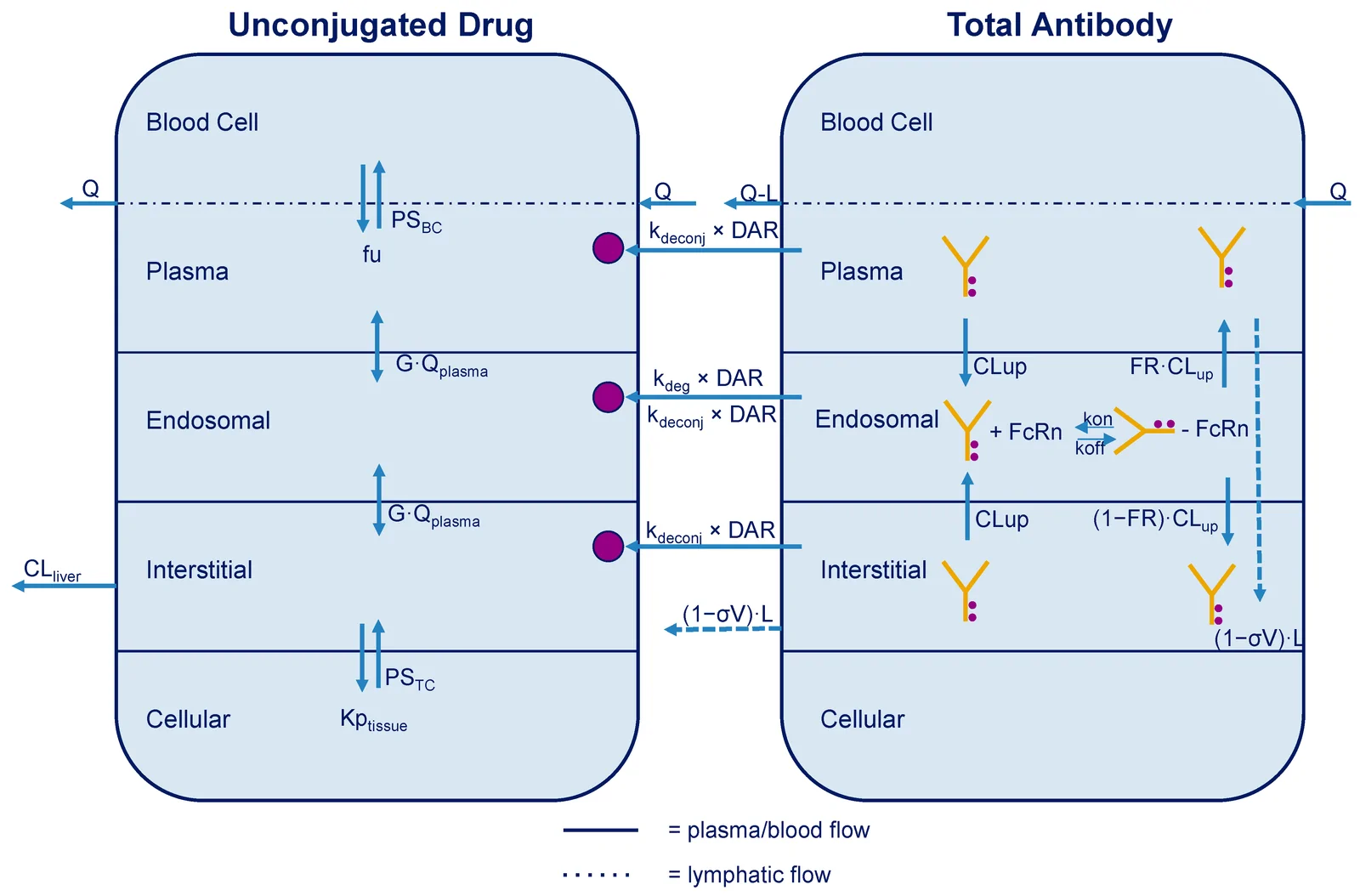
Tissue-level sub-compartment structure of the ADC PBPK model for (**left**) unconjugated drug payload and (**right**) total antibody within a representative tissue and its sub-compartment linked by permeability- and flow-limited transport like permeability–surface area products (PS) (PS_BC_, PS_TC_, G·Qplasma), partitioning (Kp, _tissue_), and hepatic clearance (CL_liver_) for the payload, as well as by pinocytotic uptake (CL_up_), FcRn-mediated recycling/transcytosis (kon/koff, FR·CL_up_), and two-pore convective transport (σV, σIS, L) for the antibody. Purple markers show payload entering the small-molecule panel via deconjugation (k_deconj_ × DAR) and degradation (k_deg_ × DAR), linking the two models. CL_liver_, hepatic clearance; CL_up_, pinocytotic uptake clearance; DAR, drug-to-antibody ratio; FcRn, neonatal Fc receptor; FR, fraction of FcRn-mediated complex recycled; k_deconj_, deconjugation rate constant; k_deg_, degradation rate constant; k_on_ and k_off_, association and dissociation rate constants for FcRn binding; Kptissue, tissue-to-plasma partition coefficient; L, lymph flow; PS, permeability–surface area product; σ*V* and *σ*IS, vascular and interstitial reflection coefficients. Adapted from the tissue-level PBPK framework of Chang and Shah [51].

**Table 1:** Details about the components of USFDA-approved ADC formulations (Drugs@FDA) [18]. Abbreviations: BCMA, B-cell maturation antigen; CD, cluster of differentiation; DAR, drug-to-antibody ratio; DM1 and DM4, maytansinoid payloads; DXd, exatecan derivative payload; FRα, folate receptor alpha; HER2, human epidermal growth factor receptor 2; MMAE, monomethyl auristatin E; MMAF, monomethyl auristatin F; PAB, para-aminobenzyl; PBD, pyrrolobenzodiazepine; SN-38, 7-ethyl-10-hydroxycamptothecin; TF, tissue factor; TROP2, trophoblast cell-surface antigen 2; USFDA, United States Food and Drug Administration.

Name	Antibody	Payload	Linker	Conjugation	DAR
Belnatamab mafoditin	Anti-BCMA	Monomethyl auristatin F (MMAF)	Non-cleavable	Partial cysteine alkylation	4
Brentuximab vedotin	Anti-CD30	Monomethyl auristatin E (MMAE)	Valine–citrulline	Partial cysteine alkylation	4
Datopotamab deruxtecan	anti-TROP2 IgG1	Topoisomerase I inhibitor	Tetrapeptide	Cleavable tetrapeptide	4 (approx.)
Enfortumab vedotin	Anti-nectin-4	MMAE	Valine–alanine	Cysteine alkylation	3.8
Gemtuzumab ozogamicin	Anti-CD33	N-acetyl calicheamicin	Cleavable hydrazone and disulfide	Lysine–amide coupling	2–3
Inotuzumab ozogamicin	Anti-CD22	N-acetyl calicheamicin	Cleavable hydrazone and disulfide	Lysine–amide coupling	6
Loncastuximab tesirine	Anti-CD19	Pyrrolobenzodiazepine (PBD) dimer	Valine–alanine	Partial cysteine alkylation	2.3
Mirvetuximab soravtansine	Anti-FRα	DM4 (maytansine derivative)	Disulfide	Lysine–amide	3.5
Polatuzumab vedotin	Anti-CD79b	MMAE	Valine–citrulline	Partial cysteine alkylation	4
Sacituzumab govitecan	Anti-TROP2	SN-38	Lysine–PAB and carbonate	Cysteine alkylation	7.6
Telisotuzumab vedotin	Anti-c-Met	MMAE	Valine–citrulline	Cysteine alkylation	3
Tisotumab vedotin	Anti-TF	MMAE	Valine–citrulline	Cysteine alkylation	4
Trastuzumab deruxtecan	Anti-HER2	Dxd (exatecan)	Glycine–glycine–phenylalanine–glycine	Cysteine alkylation	3.8
Trastuzumab emtansine	Anti-HER2	DM1 (Mayatansine derivative)	Non-cleavable	Lysin–amide	3.5

**Table 2:** Summary table showing effect of linkers on physiological and PK properties.

Linker	Cleavable Bond/Type	Physiological Property Affected	Pharmacokinetic Effect
Arylsulfate (sulfatase-cleavable) [7]	Enzyme-cleavable (sulfatase)	High plasma stability; tumor-selective cleavage	Stable >7 days in mouse plasma; Val-Ala/Val-Cit hydrolyze within 1 h
Carbonate (Trodelvy) [27]	Acid-cleavable	Insufficient plasma stability	t_1_/_2_ = 36 h; limits use with highly cytotoxic payloads
DiMe-SS vs. Me-SS disulfide [28]	GSH-cleavable	Controls rate of intratumoral self-cleavage and payload release	DiMe-SS: 42.1 vs. 19.1 nmol/L MMAE in tumor; PAB addition further improves to 87.1 nmol/L
Hydrazone (Mylotarg) [29]	Acid-cleavable	Insufficient plasma stability; non-specific hydrolysis	t_1_/_2_ = 2 days in human/mouse plasma; hepatotoxicity risk
MD linker (1,3-dioxane maleimide) [30]	Non-cleavable	Increased hydrophilicity via ring-opening hydrolysis	Only 3% plasma degradation in 120 h; improved circulatory stability
Methyl/cyclobutyl disulfide [31]	GSH-cleavable	Determines intratumoral catabolite identity and activity	Active PBD delivered at 1.0–2.0 nmol/L in tumor; cyclopropyl analogue yields only inactive catabolite
N-phenyl maleimide [32]	Non-cleavable	Stabilized thiol conjugation via thiosuccinimide hydrolysis	Payload release reduced to 15% vs. 45% (N-alkyl) over 7 days in mouse plasma
PEGylated linker (PEG0–PEG12) [33]	Various	Hydrophilicity directly reduces non-specific hepatic uptake	PEG12: clearance 7.3 mL·kg/day vs. >46.3 mL·kg/day (PEG0); improved systemic exposure
Polysarcosine (PSAR) linker [34]	Enzyme-cleavable (β-glucuronidase)	Superior hydrophilic shielding vs. PEG; supports DAR = 8	Clearance 38.9 vs. 47.3 mL/day/kg (PEG); reduced aggregation at high drug loading
Pyrophosphate diester [35]	Enzyme-cleavable (phosphatase)	High hydrophilicity; reduces aggregation of lipophilic payloads	Stable >7 days in plasma; solubility >5 mg/mL; improves distribution of lipophilic payloads
Silyl ether [36]	Acid-cleavable	Improved plasma stability; supports cytotoxic payloads	t_1_/_2_ > 7 days in human plasma; suitable for MMAE delivery
Succinimidyl 4-(N-maleimidomethyl) cyclohexane-1-1carboxylate (maleimide) [37]	Non-cleavable	Hydrophobic; susceptible to retro-Michael elimination	38% plasma degradation in 120 h; 29% DAR loss in 7 days in mice; reduced exposure and efficacy
Triglycyl CX linker [38]	Cathepsin-cleavable	Ces1C-resistant; equivalent human plasma stability to SMCC	t_1_/_2_ ~9.9 days, CL 0.7 mL/h/kg; 50-fold higher therapeutic index vs. SMCC-DM1
Val-Ala dipeptide [39]	Cathepsin-cleavable	Better hydrophilicity than Val-Cit; reduced aggregation	Lower plasma aggregation (vs. 1.80% for Val-Cit) at DAR ~7
Val-Cit dipeptide [40]	Cathepsin-cleavable	Degraded by plasma carboxylesterase 1C (Ces1C) in mice	Preclinical metabolic instability; species-specific PK liability

**Table 3:** The four tissue sub-compartments of an ADC PBPK model, their physiological basis, the processes they represent, and the parameters required to specify them [41,47,48,49,50,51,52,53,54].

Sub-Compartment	Physiological Basis	Process Represented	Parameters Required
Vascular	Blood volume ≈5.5 L, ≈55% plasma; cardiac output ≈5 L/min; albumin 30–50 g/L and endogenous IgG 8–18 g/L generate the oncotic pressure opposing filtration	Arterial delivery and venous drainage; oncotic balance setting the rate of extravasation	Organ blood flow (Q); venous flow (Q–L); plasma fraction; blood-to-plasma ratio
Endothelial	Permeability set by capillary type: continuous in muscle, heart and lung; fenestrated in kidney, gut and endocrine glands; sinusoidal in liver, bone marrow and spleen. CSF IgG ≈500-fold below plasma	Fluid-phase endocytosis and catabolism, with FcRn binding rescuing Fc-containing species from lysosomal degradation	Organ-specific permeability; endosomal uptake rate; FcRn affinity at pH 6.0 and 7.4; recycled fraction
Interstitial	Interstitial fluid ≈12 L, of which ≈6 L is accessible to protein; lymph flow ≈1.5 mL/min; IgG in lymph ≈30% of plasma	Convective extravasation through small and large pores, lymphatic return, and receptor-mediated uptake by parenchymal cells	Permeability–surface area products; osmotic reflection coefficients for each pore population; protein-accessible volume fraction
Cellular	For a trastuzumab–maytansinoid ADC, internalization t½ 6–14 h, antibody proteolysis 18–25 h and payload efflux 44–73 h; up to 45% of internalized antibody recycled for an EGFR-targeted ADC	Sorting between recycling and lysosomal degradation, and payload liberation, within the endo-lysosome for cleavable linkers or requiring full backbone proteolysis for non-cleavable ones	Internalization and degradation rate constants; recycled fraction; payload efflux rate

## Data Availability

No new data were created or analyzed in this study. Data sharing is not applicable to this article.
